# Promoting life's essential 8 to advance global adolescent cardiovascular health

**DOI:** 10.1016/j.ijcrp.2025.200507

**Published:** 2025-09-01

**Authors:** Jason M. Nagata, Nathan D. Nguyen, Christiane K. Helmer, Jennifer H. Wong, Abubakr A.A. Al-shoaibi, Holly C. Gooding

**Affiliations:** a Department of Pediatrics, University of California, San Francisco, 550 16th Street, 4th Floor, Box 0503, San Francisco, CA, 94143, USA; b Division of General Pediatrics and Adolescent Medicine, Department of Pediatrics, Emory University School of Medicine, Children's Healthcare of Atlanta, 2015 Uppergate Dr, Atlanta, GA, 30322, USA

**Keywords:** Adolescent, Global health, Prevention

Dear Editor,

We appreciate the thoughtful letter, "From classroom to clinic: Social determinants of adolescent cardiometabolic health in the Global South," by Lacsa [1]. While our study [2] focused on American early adolescents, we agree with their call for more global research on adolescent cardiovascular health (CVH), especially for low- and middle-income countries. Roughly 90 % of adolescents reside in low- and middle-income countries, but there is limited data to inform evidence-based programs and address the specific needs of this population. Concurrently, the influence of structural and social inequalities on cardiometabolic health is increasingly clear [3]. An analysis of the 2019 Global Burden of Disease Study revealed a rising burden of cardiovascular disease among youth and young adults in low- and middle-income countries [4]. While many risks, such as poor nutrition and physical inactivity, are rooted in structural inequities, they remain modifiable through targeted interventions. Addressing these disparities will require further research and global collaboration to develop evidence-based solutions.

The Life's Essential 8 (LE8) score, released by the American Heart Association, provides a comprehensive framework for assessing CVH that includes nutrition, physical activity, nicotine use, sleep, body mass index, blood lipids, blood glucose, and blood pressure, and acknowledges their social and structural determinants [3]. Recent studies have assessed LE8 scores in global contexts [5], but there is limited data on adolescent CVH in low- and middle-income country settings. Targeted, locally relevant interventions like those proposed by Lacsa to improve adolescent CVH will be necessary to reach the American Heart Association 2030 Impact Goal of improving CVH worldwide [6].

## CRediT authorship contribution statement

**Jason M. Nagata:** Conceptualization, Writing – original draft, Writing – review & editing. **Nathan D. Nguyen:** Writing – original draft, Writing – review & editing. **Christiane K. Helmer:** Writing – original draft, Writing – review & editing. **Jennifer H. Wong:** Writing – original draft, Writing – review & editing. **Abubakr A.A. Al-shoaibi:** Writing – original draft, Writing – review & editing. **Holly C. Gooding:** Conceptualization, Writing – review & editing.

## Funding

J.M.N. was funded by the National Institutes of Health (K08HL1549350 and R01MH135492) and the Doris Duke Charitable Foundation (2022056).
